# Unveiling the potential of banana (*Musa* spp.) improvement through genetic manipulation: current trends and future implications

**DOI:** 10.1080/15592324.2026.2656013

**Published:** 2026-04-08

**Authors:** Utpal Das, Shiva Sai Prasad, Tanushree Sahoo, Sourav Paramanik, Amrita Halder, Poonguzhali S.

**Affiliations:** a Department of Horticulture and Food Science, VIT School of Agricultural Innovations and Advanced Learning (VAIAL), Vellore Institute of Technology, Vellore, India; b Department of Agriculture, Koneru Lakshmaiah Education Foundation, Green Fields, Vaddeswaram, India; c Scientist (Fruit Science), ICAR-Indian Institute of Horticultural Research, Central Horticultural Experiment Station (CHES), Bhubaneswar, India; d Department of Genetics and Plant Breeding, VIT School of Agricultural Innovations and Advanced Learning (VAIAL), Vellore Institute of Technology, Vellore, India

**Keywords:** *Musa* spp., CRISPR/Cas9, genetic engineering, abiotic stress, diseases resistances

## Abstract

Banana (*Musa* spp.) is a globally important fruit crop and a staple food for millions of people. However, its narrow genetic diversity and clonal propagation make it highly vulnerable to pests, diseases, and abiotic stresses. Genetic improvement is limited by sterility, and triploid plants are reproduced clonally in most cultivated varieties, preventing traditional breeding based on genetic advancements. Transgenesis and gene editing are among the genetic engineering techniques used to increase yield, improve quality, and enhance resilience. Advances in Agrobacterium-mediated transformation and CRISPR/Cas tools have enabled the development of bananas with enhanced resistance to Fusarium wilt tropical race 4 (TR4), Black Sigatoka, and bacterial wilt, along with increased provitamin A content, longer shelf life, and reduced postharvest losses. DNA-free genome editing provides a promising approach to overcoming certain regulatory barriers and enhancing public acceptance. While challenges such as genotype-specific transformation efficiency, regulatory hurdles, and public perception persist, genetic manipulation holds the potential to both preserve and improve global banana production. This review synthesizes recent progress, key targets, and future prospects for the genetic improvement of banana.

## Introduction

Bananas (*Musa* spp.) are among the world's leading fruit crops and have been cultivated since the beginning of formal agriculture. In India, banana ranks second among the most important fruit crops in the country, next to the mango and contributes a significant percentage of the country's fresh fruit production. Scientifically, banana is a large herbaceous perennial monocot that matures much faster than most fruit crops and produces fruit on a yearly round basis. The *Musa* genus is highly valued globally due to the commercial importance and nutritional benefits of cultivated varieties. Bananas and plantains are vital for livelihoods and economic stability, especially in regions with unstable regimes caused by poor environmental conditions.^1^ Banana acts as the dominant crop of Uganda occupying about 40% of arable area acting as a staple food.^2^ *Musa* species are relied upon directly and indirectly by many households worldwide involved in banana and plantain production, among other products. In 2011, banana cultivation covered about 10.6 million hectares with an average yield of nearly 13.6 tons per hectare.^3^ By 2017, global production reached 114 million tons annually.^4^ *Musa* species are cultivated in more than 135 countries, with the highest production taking place in the humid tropical and subtropical locations.^5^ Even though India and Brazil are considered countries with the highest banana production, their exports to international markets remain minimal. The warm, humid climate with consistent moisture supply and low wind facilitates its optimal growth. While primarily tropical plants, bananas have adapted to various climate conditions, including wet and dry tropical zones and even some dry subtropical areas. However, banana production faces significant challenges from abiotic and biotic stress. Major abiotic stresses include drought, temperature extremes and salinity, while biotic stresses involve fungi and bacteria, all posing severe constraints. Viral pathogens such as banana virus X (BVX), banana bunchy top virus (BBTV), banana streak virus (BSV), banana bract mosaic virus (BBrMV), banana mild mosaic virus (BgMV) and cucumber mosaic virus (CMV) significantly impact cultivation.^6^ Losses from insects and nematodes were once considered as severe as those from pathogens. However, research on abiotic stresses in bananas has received less attention than biotic stresses. The *Musa* genus includes around 1,000 dessert and culinary cultivars of banana and plantain, all of which originated through hybridization between two wild diploid ancestors, *Musa acuminata* and *Musa balbisiana*. Morphological data reveal significant variation within *Musa* and provide clear evidence of different genome compositions. Nonetheless, phenotypic characterization of several traits, such as stress tolerance, remains challenging in controlled environments due to the plant's large size and long lifecycle. Other barriers to banana improvement include natural parthenocarpy, high levels of sterility, and a protracted growth cycle, which collectively limit the efficacy of traditional hybridization efforts across all breeding objectives. Additional constraints include low seed viability, irregular meiotic behavior, and highly variable genomic arrangements.^7^ Modern plant science offers DNA markers that are invaluable for describing germplasm across numerous plant species. Recent rapid advances in genotyping methods, such as diversity arrays technology (DArT) and next-generation sequencing-based markers, have been used in *Musa* research to facilitate genetic studies and crop improvement.^8^ Due to their codominant nature, reproducibility, and abundance in plant genomes, SSR markers remain effective for assessing genetic diversity in many plant species, including bananas.^9^^,^^10^ The success of breeding programs depends largely on maintaining genetic variability. To date, significant improvements have been achieved through conventional cross-breeding. There is therefore an ongoing need to develop better strains with stable yields and favorable fruit qualities suitable for various environmental stresses. This paper highlights the current state of banana genetic improvement and examines modern techniques developed to increase banana production.

### Taxonomy classification of *Musa* cultivars

The genus *Musa* can be traced back to the root “mouz”, which in Arabic means that the plant's name was borrowed. This has been suggested as the Arabicized name later given to Antonius *Musa* (63 BC–14 AD), personal physician to Emperor Augustus. Most of the modern *Musa* cultivars are largely sterile with parthenocarpy fruits and are believed to have formed through natural hybridization between the wild species *Musa acuminata* (AA) and *Musa balbisiana* (BB), both of which have a diploid chromosome number (2n = 2× = 22).^11^ Due to this inherent sterility and the long growth cycle of the plant, traditional hybridization is significantly restricted. Consequently, somatic hybridization and recombinant DNA technology have become essential tools for the genetic improvement of banana, regardless of the specific breeding target. Most commercial varieties are triploid (2n = 3× = 33) and consist of genome combinations of AAA, AAB, or ABB. Sweet bananas are derived from homogenous triploid (2n = 3× = 33) hybrids with the AAA genome that originated from *Musa acuminata* through interspecific hybridization. Most cooking bananas (plantains, AAB and other ABB subsets) are heterogenous triploid hybrids (2n = 3× = 33), resulting from crosses between *Musa acuminata* (AA) and diploid *Musa balbisiana* (BB).^12^

*Musa* species are not easily distinguished by sex because vegetative reproduction is common and natural hybrids are widespread. Currently, this genus is divided into five sections, namely *Eumusa* (2n = 2× = 22), *Rhodochlamys* (2n = 2× = 22), *Australimusa* (2n = 2× = 20), *Callimusa* (2n = 2× = 20), and *Ingentimusa* (2n = 2× = 14).^13^ However, *Ingentimusa* was later merged into *Musa* after^14^ challenged Argent's suggestion that a separate section was necessary. Instead, *Musa ingens* was shown to be more closely related to other species within the *Musa* section. Recent molecular studies concluded that the genus *Musa* L. is limited to only two sections. Based on molecular phylogenetic evidence,^15^ argues that intraspecific comparisons of phylogenetic data require a revaluation of the infrageneric taxonomy of *Musa* L. As a result, section Rhodochlamys was combined with section *Musa*, while sections *Australimusa* and *Ingentimusa* were merged into a large section *Callimusa*. The other genus in *Musaceae* is *Ensete*, which includes the Ethiopian banana (*Ensete ventricosum*). This species is sometimes cultivated as a food crop in parts of East Africa. Initially, Cheesman recognized 21 *Musa* species and 25 *Ensete* species, but subsequent taxonomic research has identified more than 80 *Musa* species, depending on the authority.

### Genetic diversification and domestication of *Musa*

Bananas are among the first crops domesticated by farmers and now spread throughout the subtropics. They quickly became a popular starchy staple in regions like Africa and Asia, and in many other countries, they turn into a cash crop when cultivated as dessert bananas. Ordinary bananas and plantains are grown by farmers in the humid tropical and subtropical areas of the Americas, Africa, Asia, and even extend to Europe and Australia. About 1,000 banana cultivars, including landraces, have been identified worldwide. Cultivated bananas and plantains are seedless and parthenocarpy, whereas their wild counterparts produce seeded fruits. The current domestication scenario suggests that most banana cultivars originated when early farmers selected wild relatives that naturally produced mutants capable of parthenocarpy and were propagated vegetatively.^16^^,^^17^ Modern banana breeders believe this is accurate. These early selections likely favored hybrid varieties that were diploid and triploid, with sweet flesh and few seeds.^18^ Most modern dessert bananas with genomes such as AAA, AAB, and ABB are triploid with 2n = 3× = 33.^19^ There are also diploid AA and AB lines as well as tetraploids (2n = 4× = 44) with genomes including AAAA, AAAB, AABB, and ABBB, as well as some of these are starchy plantains. Natural hybridization and mutations over the past two to three centuries have created a wide variety of banana types. Recent research on over 300 *Musa* acuminata genotypes shows that most cultivars descend from two subspecies, *Musa acuminata banksii* and *Musa acuminata errans*.^20^

### Genetic mapping and trait inheritance of *Musa*

A broad understanding of the genetic basis of a specific agronomic trait is crucial for developing a successful breeding program. However, only a few empirical genetic studies have been conducted on tropical bananas due to their sterility. At the phenotypic level, in both suckers, dwarfism and pseudo-stem waxiness, these traits are controlled by a single recessive gene, with incomplete penetrance and variable expressivity influencing which traits manifest.^21-23^ Bunch orientation and fruit parthenocarpy seem to be governed by an oligogenic system. Using parent-offspring regression, found that bunch weight, the number of fruits, and fruit length exhibit moderate to low heritability in tetraploid plantain bananas of 3×–2× crosses, indicating limited effectiveness of selection based solely on phenotypes for triploid parents and the necessity of progeny testing.^24^ According to Nyine et al.^25^ demonstrated positive correlations between high growth and yield traits; plant height at flowering had the strongest positive correlation with plant girth at 100 cm above ground and was associated with the tallest sucker at flowering. Plant girth at 100 cm also positively correlated with bunch weight at full maturity, the number of finger units, and the maximum height of the tallest sucker at flowering.^26^ These findings support the development of genomic selection models using easily measurable, highly correlated traits, including the height of the tallest sucker at flowering, which are otherwise difficult to phenotype. In *Musa acuminata*, cytogenetic studies show that chromosomal pairing is regular in subspecies, but regular pairing, including multivalent and univalent arrangements, occurs in hybrids formed between these subspecies.

Mapping chromosomes is a key strategy for identifying molecular markers linked to major or minor loci controlling individual traits or quantitative trait loci (QTL). These markers can help identify the genes responsible for these features, facilitating genetic improvement of selected lines. The isolated genes can be introduced into different cultivars via recombinant DNA technology or MAS, allowing preferred plant phenotypes to emerge in segregating populations. *Musa* genetic mapping is still in early stages; however, linkage mapping with 90 loci in an F_2_ population of *Musa acuminata* produced the first linkage map using two genetically divergent genotypes, *SF265* (AA) and *Banksii* (AA). This study employed isozyme, RFLP, and RAPD markers. The heterogeneity in the genetic backgrounds of the grandparental lines required sufficient polymorphisms to construct accurate linkage maps. As a result, 15 linkage groups were obtained, corresponding to the haploid chromosome number in *Musa*. Challenges such as low viability, high costs, spatial limitations, and disease impacts hinder mapping populations. To address these issues, alternative methods are being explored to study *Musa* genetics. One approach involves using a different culture to produce haploid banana plants that may spontaneously develop double chromosomes. Another valuable resource for linkage mapping is the double-haploid (DH) plant. Genetic mapping can be efficiently performed via tissue culture when large numbers of DH plants are produced from a single, recombining heterozygous pollen source. Unlike conventional methods involving large segregating populations such as F_2_, F_3_ (F_2_-derived), backcross, near-isogenic lines (NILs), or recombinant inbred lines (RILs), association mapping offers a modern approach.^27^ It utilizes genetically diverse genotypes from natural populations and leverages linkage disequilibrium between markers and targeted loci to evaluate the relationship between markers and traits.

### Molecular cytogenetics and genomic editing of *Musa*

**Figure 1.**
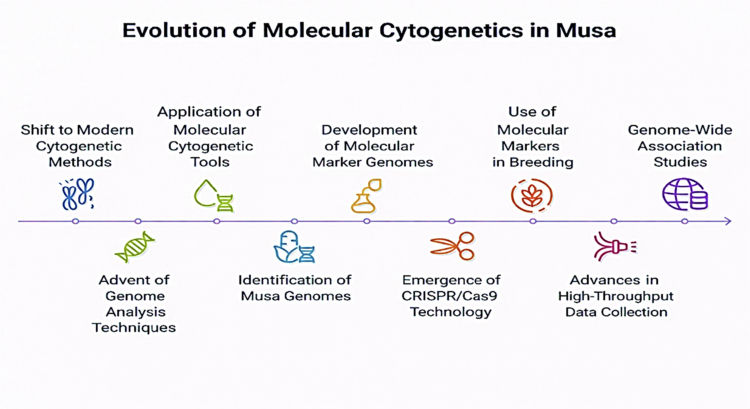
Presenting the evolution of molecular cytogenetics in *Musa* cultivars.

The overview of the past two decades is marked by a major shift towards modern cytogenetic methods for studying chromosomes, replacing traditional cytogenetic research. With the advent of genome analysis techniques, the study of plant genomes has become much more detailed and precise. These methods, mainly including hybridization or PCR-based marker analysis, fluorescent and genomic in situ hybridization (FISH and GISH), DArT, and high-resolution DNA melting analysis (HRM), have proven successful in analyzing the genetic makeup and ploidy structure of *Musa accessions* (Figure 1). These molecular cytogenetic tools were first applied to identify the genome in *Musa* by Osuji et al.^28^ Later, Dolezel et al.^29^ used GISH and FISH to identify chromosomes associated with different genomic compositions. Overall, these techniques help distinguish the four genomes (A, B, S, and T) found in banana cultivars and hybrids by using specific fluorochromes. In situ hybridization studies have also revealed the location of middle repetitive sequences within the *Musa* genome, revealing both repetitive DNA sequences and single-copy sequences. Based on competitive PCR, empirical studies also show that more than 1% of the *Musa* genomes consist of families of repetitive DNA sequences. According to Osuji et al.^28^ reported significant differences between the repetitive A and B genomes. Valarik et al.^30^ cloned and characterized several of these repetitive sequences, identifying those not associated with rDNA or retroelement families in the centromeric region of the *Musa* chromosomes.

Advances in molecular biology have led to the development of emerging technologies, including molecular marker probes targeting specific traits, which have become essential in accelerating crop improvement programs. Notably, CRISPR/Cas9 technology has become prominent in efforts to develop disease-resistant banana cultivars. These advances have the potential to address key challenges in banana cultivation, such as virus resistance and disease management. A landmark study demonstrated that CRISPR/Cas9 can be used to target and knock out endogenous banana streak virus (eBSV) sequences integrated into the B genome, effectively preventing the activation of the virus into its infectious form.^31^ Its effectiveness in genome editing bananas has been confirmed by recent studies, which have demonstrated successful modifications.^32^ Furthermore, Hu et al.^33^ found that the absence of CRISPR/Cas-mediated intervention at the *MaACO1* gene delays the postharvest shelf-life of bananas. Additionally, this method has provided early insights into the carotenoid cleavage pathway in bananas. According to Awasthi et al.^34^ both protoplasts and embryogenic cells are suitable for transgene-free editing of the banana genome. These advances have the potential to address key challenges in banana cultivation, such as virus resistance and disease management. A molecular marker is a specific segment of DNA spread throughout the genome that should not be considered a gene, although it serves as a tag to locate a particular trait on a chromosome. Once genetic markers are identified, they can be incorporated into breeding programs, as they enable the prediction of phenotypic traits based on the presence or absence of specific alleles. One key benefit of this approach is that it facilitates the analysis of complex, polygenic traits.^35^ Molecular markers have been widely used for germplasm characterization in *Musa*, genetic diversity studies, DNA fingerprinting, assessing genetic fidelity, and locating candidate genes. As described by Igwe et al.^36^ various molecular markers have been used to investigate different genetic aspects of bananas. Table 1 summarizes the molecular markers already used in banana genomics studies, along with their main applications. Sequence tagged microsatellite site (STMS) markers are highly valuable for identifying clones in the International *Musa* Germplasm Collection, now maintained at the Catholic University of Leuven in Belgium, as well as for identifying interspecific hybrids. These markers have also been used to explain intraspecific genetic variation, especially in differentiating the A and B subgenomes, understanding evolutionary and phylogenetic trends, and serving as foundational tools for marker-assisted selection (MAS) in breeding programs. Advances in high-throughput data collection, particularly single-nucleotide polymorphisms (SNPs) and small insertions/deletions (indels), provide enhanced specificity and sensitivity in identifying alleles and haplotypes.^37^ Recently, genome-wide association studies (GWAS) have represented new opportunities for identifying candidate genes associated with the trait of interest. However, this approach has not yet been applied to a vegetatively propagated crop.^38^ In one study, 13 potential genomic regions were identified containing genes associated with the seedless phenotype-parthenocarpy alongside female sterility.^38^ These findings deepen our understanding of the genetic basis of such traits and could accelerate genetic improvement in bananas.

**Table 1.** Molecular markers used in different banana improvement programs.

Application	Marker(s)	Reference
Genetic variability and diversity studies	RAPD, AFLP, SSR, EST-SSR, ISSR, DArT, DAMD, MSAP	[25,39-44]
Genetic stability studies	RAPD, ISSR	[45]
Germplasm characterization and phylogeny study	Isozymes, RFLP, RAPD, SSR, EST-SSR, ISSR	[10,46,47]
Linkage analysis and map construction	Isozyme, AS-PCR, RAPD, RFLP, AFLP, SSR, DArT	[48,49]
Genome-wide association study (GWAS)	SNP	[38,50-52]

Recently, major genome sequencing projects such as the European Arabidopsis Genome Sequencing Project, the International Human Genome Project, and the International Rice Genome Sequencing Project set the precedent by sequencing the banana genome (*Musa acuminata*).^53^ In 2001, this project aimed to sequence 550–600 Mb of DNA in each haploid genome across 11 chromosomes within four years, involving scientists from 11 countries, including both profit and nonprofit institutions (Global *Musa* Genomics Consortium). Dolezel et al.^29^ estimated the genome size of *Musa* as 550 Mb for *M. balbisiana* and 600 Mb for *M. acuminata*. These estimates were further refined by Bartos et al.^54^ who included additional *Musa* and *Ensete* species from all parts of the genus. The sequencing process encompasses both coding and noncoding regions, such as unique and repetitive DNA pseudogenes, separated genes, and transposons, as well as the arrangement of these components on chromosomes, centromeric and telomeric regions, and the total number of open reading frames (genes). Analysis of 6,252 end sequences from *Musa* bacterial artificial chromosome (BAC) clones revealed a genome-wide GC content of 47%. Further analysis of BAC-end sequences suggests an average of roughly one coding gene every 6.4 to 6.9 kb in the genomic DNA, with lower gene density in regions rich in transposable elements.

### Advances in *Musa* genome sequencing and pan-genomic frameworks

The quality of genomes is essential for the efficiency of genome editing technologies, including CRISPR/Cas9, as it enables accurate target identification and reduces off-target effects. The sequencing of *Musa acuminata* (A-genome) haploid genome (~523 Mb) marked the first breakthrough in banana genomics, providing the initial comprehensive understanding of the 11 chromosome sets.^55^^,^^56^ This was followed by further advancements in coding and noncoding elements, revealing a ratio of about one coding gene to 6.4–6.9 noncoding genes. Recent progress in banana breeding has shifted from relying on a single reference genome to constructing comprehensive pan-genomes capable of capturing large structural variations (SVs), transposable elements, and gene presence‒absence variations (PAVs) across different cultivars.^57^^,^^58^ Massive sequencing of over 300 *Musa acuminata* genotypes has provided invaluable insights into the ancestry of current banana varieties, with most originating from specific subspecies such as *M. acuminata banksii* and *M. acuminata errans*. High-resolution genomic data now allow precise differentiation of A, B, S, and T genomes, which is crucial for exploring the complex genetic makeup of heterologous triploids (AAA, AAB, ABB).^17^^,^^59^ Additionally, these genomic tools have identified key regions associated with vital agronomic traits, including female sterility and the seedless (parthenocarpic) phenotype. Through the combined use of single-nucleotide polymorphisms (SNPs) and genome-wide association studies (GWASs), researchers are pinpointing candidate genes related to stress resilience, such as the *MaAGPase* and *MaAQP* gene groups linked to drought tolerance.^60^ This provides a solid foundation for applying genome editing technologies to improve stress resilience in bananas.

### Enhanced propagation for stress resistance

Current climate change dynamics are causing major disruptions in agro-ecosystems by altering abiotic factors such as light intensity, water availability, salinity, temperature, nutrient balance, oxidative stress, and hypoxia. These environmental changes negatively impact banana yield and fruit quality.^61^ Among these variables, drought, salinity, and low temperature are particularly damaging to banana growth and development, often leading to significant reductions in crop productivity. According to Ravi and Vaganan,^62^ abiotic stresses such as drought, salinity, temperature extremes, and flooding can result in crop yield reductions ranging from 65% to 87%, depending on the species and severity of stress. They further indicated that ongoing climate change is expected to intensify these stresses, thereby posing a serious threat to global food availability and long-term food security.

#### Drought tolerance

According to Singh^63^ drought is a highly diverse phenomenon, making it difficult to define and measure. The working definition suggests that drought indicates a shortage of water supply in terms of quantity and its distribution over a crop's life cycle, both in terms of amount and timing, to prevent the full genetic yield potential from being realized. Globally, drought affects approximately 28% of soils.^64^ In banana cultivation, drought has become the primary abiotic stress among all environmental factors negatively impacting plant growth and development. Several studies highlight that soil moisture deficit stress affects banana growth. For example, Ravi and Vaganan^62^ report that drought during the vegetative stage delays foliage emergence, reducing yield and related parameters. Similarly, Ravi et al.^65^ found that water stress during the flowering stage reduces bunch weight by 18.83% to 42.07% depending on the cultivar also reduces fruit length by 11–14% and circumference by 5.75–16%. Water deficit imposed at the flowering stage significantly reduced finger diameter by 9% and decreased bunch fresh weight by 41% in banana. When water availability drops below 66%, banana yield can sharply decline as irrigation delays increase. Shortening the water deficit period during bunch emergence mostly results in smaller fruits, delayed fruit filling, longer green life, and fruit maturity bronzing. To conserve internal water and remain upright during moisture stress, banana plants close their stomata, which reduces carbon assimilation.^66^

Two major crop response strategies minimize yield loss due to drought in the crop system: (a) genetic engineering of drought-resistant varieties and (b) calibrated agronomic management. Drought-resistant genotypes experience less reduction in photosynthetic parameters and leaf area without increasing the associated lower leaf water content. In bananas, drought resistance involves two main strategies such as drought avoidance and drought tolerance.^64^^,^^66^ Dehydration avoidance is characterized by high hydration levels, achieved either by reducing transpiration or increasing water uptake.

Bananas are highly susceptible to drought, and their appearance under adverse conditions depends on the genotype. Several studies indicate that accessions with B genomes are more drought-tolerant compared to those with only A genomes.^67^ Notably, cultivars with the ABB genome complex show very strong drought tolerance compared to other genotypes.^65^ It has been hypothesized that the B genome enhances resistance to dry conditions because cultivars with high B genome content demonstrate better gas exchanges and resistance to air dryness. The physiological, biochemical, and agronomic responses of bananas to drought stress have been reported by earlier studies.^62^^,^^65^ A well-designed phenotyping protocol is essential before any breeding programs targeting these traits. According to Ravi et al.^65^ drought tolerance is most closely associated with traits like leaf emergence rate, leaf senescence rate, stomatal conductance, cell membrane stability (CMS), relative water content (RWC), and bunch yield. Several physiological characteristics, such as leaf water retention capacity (LWRC) and root shoot dry matter ratio, have been suggested as criteria for selecting drought-tolerant banana varieties. Therefore, these traits could be used as a basis of selection for drought tolerance in banana. However, since drought-induced yield loss is mainly observed as a reduction in yield, evaluating bunch yield under drought conditions should remain a primary focus in breeding. There is an urgent need to develop banana cultivars capable of overcoming abiotic stress to ensure sustainable production. Germplasms and landraces showing drought tolerance are being studied at institutions like the International Institute for Tropical Agriculture (IITA), Nigeria; the ICAR-National Research Center for Banana (NRCB), India; and the Centro Internacional de la Cana de Azúcar (CICY), Mexico. An experiment at ICAR-NRCB evaluated 112 genotypes from a core germplasm of 340 accessions for drought tolerance using LWRC measurement; selected genotypes showed promise in drought resistance and yield stability.^65^ Nonetheless, large-scale field evaluation of banana germplasm is limited by the long time required for maturation, making drought condition studies challenging.

**Figure 2.**
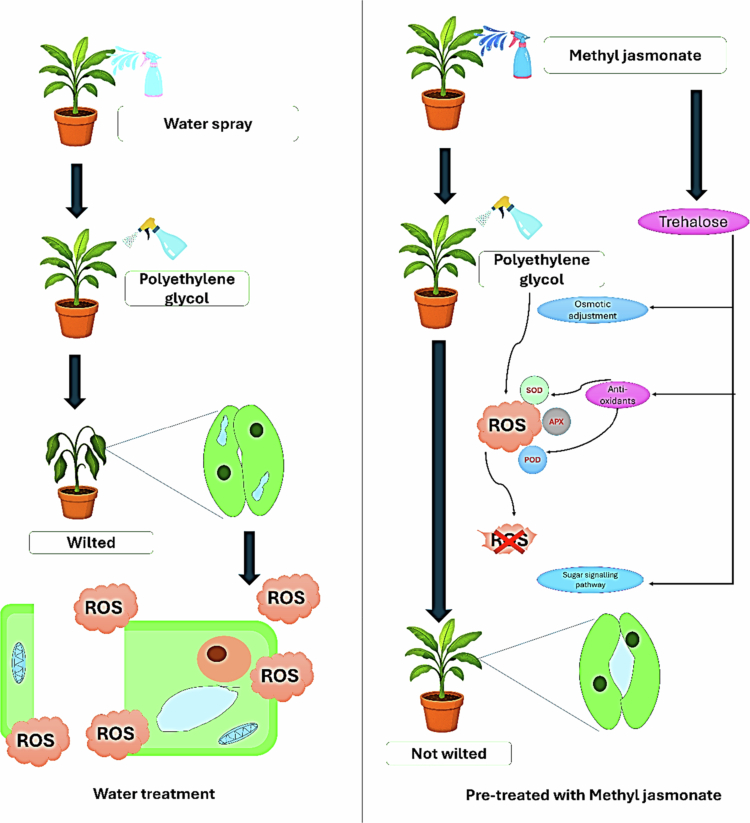
Effect of methyl jasmonate spray in alleviating the stress tolerance; a. Under the drought condition, the plant experiences ROS (Reactive oxygen species), causing water loss and cell damage; b. Prophylactic spray of methyl jasmonate will nullify the effect of ROS by the antioxidants and maintain the turgidity of stomata.

Developing drought-tolerant banana germplasm through a multidisciplinary approach is recommended. *In vitro* methods, such as tissue culture, provide a way to isolate genotypes with improved drought tolerance. Drought responses were evaluated in four *Musa* cultivars by culturing shoot tip–derived plantlets on media supplemented with polyethylene glycol (PEG) to simulate osmotic stress. Starch accumulation was assessed as a physiological indicator associated with drought avoidance mechanisms. Similarly, pretreated *Musa* acuminata cv. *Berangan* shoot tip cultures were treated with *methyl jasmonate* (MeJA) before water stress with PEG (Figure 2). Said et al.^68^ found that low-concentration trehalose treatment can protect banana plantlets against drought, likely by increasing membrane stability during stress. Combining these methods offers the best potential for enhancing drought tolerance in bananas.

The studies above highlight tissue culture's potential to produce drought-resistant banana strains. Natural genetic variation can help identify alleles that confer higher stress resistance and be integrated into future breeding programs. Currently, over 20 candidate genes have been identified as potential markers for drought tolerance in banana. Of particular interest are the different expressions of *MaAGPase*, *MaAQP*, *MabZIP*, nonredundant *DEGs*, *MaSWEETs,* and *PYL-PP2C-SnRK2* between genomes B and A.^69^ Recent studies confirm that drought-induced expression of aquaporin genes (AQP), such as *MaPIP1;1,* is highly upregulated in drought-affected banana plants.^70^^,^^71^ Water-channel proteins (AQPs) and sugar transporters (SWEETs) facilitate changes in osmotic pressure by transporting water and sugars within cells. These genes serve as valuable molecular markers for marker-assisted breeding, which is more efficient than phenotypic selection. The success of marker-assisted selection depends on the linkage between the markers and the traits of interest.^72^^,^^73^ While traditional breeding is constrained by the biological nature of the crop, the complexity of the drought-stress response specifically necessitates high-precision molecular tools. Recombinant DNA technology allows for the targeted manipulation of osmotic regulators and stress-responsive genes, providing a more viable pathway for enhancing drought resilience than conventional selection.

#### Soil salinity

Land degraded by salinity and alkalinity covers 6.73 million hectares in India. Modern studies indicate that about a quarter of irrigated areas use groundwater that is salty or brackish, and the total area affected by salinity and alkalinity was projected to reach 11.7 million hectares by 2015.^74^ Banana, a crop sensitive to salinity, faces additional pressure from increasing water shortages, especially in arid and semi-arid regions, where plant growth and productivity can be significantly hampered by salt-affected land.^61^ Salinity impacts plants through three main stresses: (1) water stress caused by osmotic effects, (2) ionic toxicity from solutes, and (3) disruptions in mineral uptake and distribution.^75^ Elevated salinity increases NaCl levels in the root zone and markedly reduces potassium absorption, leading to slower growth, delayed flowering, and lower yields. It also affects oxidative phosphorylation and the ATP/ADP ratio in roots, limiting energy supply to other plant parts.^76^ Salt stress exerts multiple effects on the biosphere, some of which are well documented. High salinity, For instance, adversely affects protein synthesis and significantly raises free amino acids and proline levels.^77^ Although plant growth regulators are often imbalanced under saline conditions, the precise role of these hormonal disruptions in salinity-related issues is not yet fully understood. For example, the *Musa* species with the acuminate clonal group is notably sensitive to salt stress. However, specific measures and genetic factors can partially reduce this sensitivity.^77^ According to Devi et al.^78^ certain cultivars such as Nendran and Robusta exhibit high sensitivity to salinity, whereas others, including Saba, Monthan, and Karpuravalli, show greater tolerance under soil conditions with an electrical conductivity (ECe) of 4–5 dS m^−1^ and pH range of 8.1–8.5.

Salt-tolerant varieties employ multiple coping mechanisms simultaneously, including activation of the *SOS* pathway to excrete Na^+^ and upregulation of antioxidant systems, which enhances production of enzymes like APX and betaine aldehyde dehydrogenase.^79^ Major genes linked to salt tolerance include salt-exclusion proteins (*SOS1* and cation/proton antiporters of the Na^+^/H^+^ family), salt compartmentalization with the help of vacuolar H^+^ pyrophosphatase, and osmotic adjustment via pyrroline-5-carboxylate synthetase (P5CS).^80^^,^^81^ When salt-tolerant banana cultivars face environmental salinity, injury typically manifests as small necrotic spots at the leaf margins, without penetrating the leaf lamina, unlike in salt-sensitive varieties. These responses lead to decreased photosynthesis, resulting in smaller bunches with lower quality. Enhancing banana salt tolerance is achievable through systematic exploration of natural variation using direct selection or QTL mapping, although the latter is challenging in vegetatively propagated species. Tissue culture screening has been common for decades.

For example, Kishk et al.^82^ found that irradiating *in vitro* banana plants with diluted seawater and mutagens, such as DES, helped identify salt-resistant genotypes. Similarly, sodium chloride was supplemented into the culture medium to simulate salinity stress under *in vitro* conditions in banana. Despite these advances, traditional breeding of perennial fruit trees remains labor-intensive and complex. The stable pathway network genes can increase salinity tolerance in transgenic plants. Recently, the gene *MaROP5g*, which is highly expressed under salt stress, has been reported to enhance tolerance by promoting root growth, maintaining membrane integrity, and regulating ion distribution.^83^ Likewise, overexpressing *MusaDHN1* led to transgenic banana lines that are more salt-tolerant due to increased proline and reduced lipid peroxidation in leaves.^84^ Overall, various salt-tolerance genes and proteins have been identified and could be integrated into breeding programs through genetic modification, introgression, or a combination of both. As traditional breeding in perennial fruit crops is slow and complex, transgenic approaches targeting stable pathway genes offer a more efficient solution. Genes such as MaROP5g and *Musa*DHN1 significantly enhance salt tolerance by improving root growth, maintaining membrane integrity, regulating ion balance, increasing proline levels, and reducing lipid peroxidation.

#### Temperature extremes

Banana cultivation heavily depends on nutrient and water availability, but experimental data show that the rate of developing new leaves and fruits mainly depends on temperature.^61^ Growth parameters, such as the rate of leaf production and the orientation and arrangement of leaves, can therefore be seen as indicators of plant development. Ideally, bunch development occurs at 21–22 °C, and temperatures above 38 °C or below 9 °C hinder growth.^62^ Leaf sunburn in banana is induced by exposure to temperatures above 38 °C, particularly under conditions of intense solar radiation. The peduncle and the uppermost hands of the bunch are especially susceptible due to their greater exposure to direct sunlight. This damage can be reduced by using protective sleeves. Conversely, low temperatures negatively impact quality and growth. Symptoms of chilling typically appear after 2–4 d and include yellowing of leaves; peel weakening results from latex coagulation, causing ducts to discolor and, in severe cases, turn gray.^62^ Bunch sleeves may also promote fruit growth in winter by buffering temperature and maintaining a uniform gradient.

According to Jangale et al.^85^ high temperatures aggravate transpiration and drought responses. Heat stress exacerbates the effect of stomatal damage through membrane ruptures and increased production of reactive oxygen species. They suggested that the size and density of stomata could serve as a feasible selection characteristic in cultivars for arid areas because small and numerous stomata provide better transpiration control than larger, fewer stomata.

Available molecular studies on temperature tolerance in banana are limited, with few published findings. According to Shekhawat and Ganapathi,^86^ identified a *bZIP* gene, *MusabZIP53*, in the banana expressed sequence tag (EST) database, and characterized it thoroughly in transgenic Rasthali plants. This research also showed that *MusabZIP53* is upregulated in native, untreated plants exposed to cold and drought stress, as well as in ABA-treated foliage and roots.^86^ It further demonstrates that, as a result of stress induction, a group of thermo-protective genes, including *HSP70*, *HSP90*, and *DNAJ* family, are concurrently upregulated, thereby contributing to thermotolerance by downregulating the corresponding microRNA (miRNA) targets.^87^^,^^88^ Such findings highlight the potential of miRNAs to act as master regulators of plant growth and development, even though they comprise only about 1% of plant protein-coding genes. Currently, as far as the authors know, no efforts have been reported to develop heat-tolerant banana varieties effectively. The urgent need for temperature-tolerant cultivars is evident to maintain maximum productivity and fruit quality in the face of modern global climate change.^89^ These insights can potentially be introduced through multidisciplinary approaches such as mutation breeding, transgenic breeding, or somatic hybridization.

### Disease resilience *via* genetic engineering

Disease resilience in the banana plant refers to its ability to resist or recover after being infected by pathogens, including fungi, bacteria, viruses, and nematodes.^90^ This resilience is supported by resistance genes that help the plant withstand major threats like Panama disease, Banana Bunchy Top, Black Sigatoka, and Banana Xanthomonas Wilt (Table 2). Current resilient building efforts involve advanced breeding technologies, genetic engineering, and phenotyping of wild relatives that can serve as sources of desirable traits.^56^ By utilizing resistance genes, a focus of recent studies and breeding initiatives, scientists are developing banana cultivars with increased resistance to emerging and re-emerging pathogens. These strategies not only help sustain crop yields but also decrease dependence on chemical control methods, promoting environmentally friendly farming practices.

**Table 2.** List of resistance genes associated with different banana diseases.

Disease	Pathogen	Resistance gene	Remark	References
Fusarium Wilt	*Fusarium oxysporum f.sp. cubense* (Foc), esp.TR4	RGA2, MaRGA08, MaRGA18, MaRGA12, MaRGA20	These are part of the NBS-LRR gene family.	[91,92]
Black Leaf Streak	*Mycosphaerella fijiensis*	RGA1, RGA2, MaSigQTL1, MaSigqtl3, *Musa*LRR	Linked with delayed leaf spotting and lower disease index.	[93]
Banana Bunchy Top	*Banana Bunchy Top Virus*	BBTV-res QTL, BanLac-1, MaBBTVR1	*BanLac-1* is a lectin gene shown to suppress BBTV replication.	[94-96]
Xanthomonas Bacterial Wilt	*Xanthomonas campestris pv. musacearum*	Pf1p, Hrap, Xa21, MaXanR1	Transgenic expression of *Pflp* & *Hrap* shows strong resistance.	[97-99]
Banana Streak	*Banana streak virus* (various species)	BSV-integration suppression loci, MaBSVR1	Problematic due to activation from the endogenous viral elements.	[31,100-102]
Burrowing Nematode	*Radopholus similis*	MaNEMR1, RGA-Nem1, RGA-Nem3	Resistance from M. balbisiana, root vigor linked to RGA clusters.	[103-105]

#### Fusarium wilt (Panama disease)

*Fusarium oxysporum f.sp. cubense* (Foc) causes Banana Fusarium wilt, a destructive soil-borne disease that spreads through the movement of infected planting materials across various regions.^106^ Externally, the disease can be identified by the gradual yellowing of older banana leaves, a characteristic beginning at the leaf margins and progressing toward the midribs. This is followed by wilting of these leaves and eventual withering of the younger leaves until the plant dies. However, these symptoms alone are not sufficient for diagnosis, as abiotic stresses and other biotic agents can cause chlorosis or wilting as well. Therefore, additional diagnostic methods are necessary. The accuracy of identification improves with internal examination of the pseudostem and rhizome, complemented by isolating the fungus from affected tissues. In pseudostem analysis, horizontal sectioning reveals characteristic reddish-to-dark-brown lesions within the leaf bases, which make up the pseudostem.^107^ Early infections in the xylem vessels appear yellow to dark red and are particularly useful for fungus isolation. If pseudostem symptoms are absent, inspecting the rhizome becomes essential. Discolored rhizomes turn yellow-dark-red internally, initially at the periphery and then spreading inward; in advanced stages, the entire rhizome may show discoloration. Pseudostem splitting is another diagnostic indicator, though it is not exclusively caused by Fusarium wilt and may result from other factors. Therefore, an accurate diagnosis of banana Fusarium wilt requires careful consideration of all symptomatology manifestations.^108^

#### Black leaf streak (black Sigatoka)

Black leaf streak (black Sigatoka) is a destructive fungal disease affecting bananas caused by the fungus *Mycosphaerella fijiensis* (*Pseudocercospora fijiensis*). The pathogen infects the epidermis of banana leaves, creating dark streaks that impair photosynthesis and cause early leaf senescence, leading to significant reductions in yield and fruit quality. Conventional breeding for resistance is impractical because over 90% of banana cultivars exhibit high sexual sterility. Therefore, genetic engineering becomes a necessary alternative.^57^ Transgenic experiments using rice chitinase genes have demonstrated a measurable reduction in disease progression and a subsequent decrease in necrotic leaf areas. The advent of the CRISPR/Cas9 genome-editing platform has transformed efforts to combat black leaf streak disease. CRISPR/Cas9 allows for precise mutation of susceptibility or virulence genes in either the pathogen or the host, enabling the development of resistant cultivars. This study was conducted on hypomatroid bananas, which primarily reproduce clonally.^56^^,^^109^ An innovative technique using preassembled Cas9 ribonucleoprotein complexes (RNPs) avoids the use of foreign DNA sequences, producing Cas9-free edited plants, a feature with regulatory and public acceptance advantages. Current research focuses on using CRISPR/Cas9 to silence genes involved in banana susceptibility to black leaf streak, offering promising potential for long-term disease control in banana production.

#### Banana bunchy top disease (BBTD)

**Figure 3.**
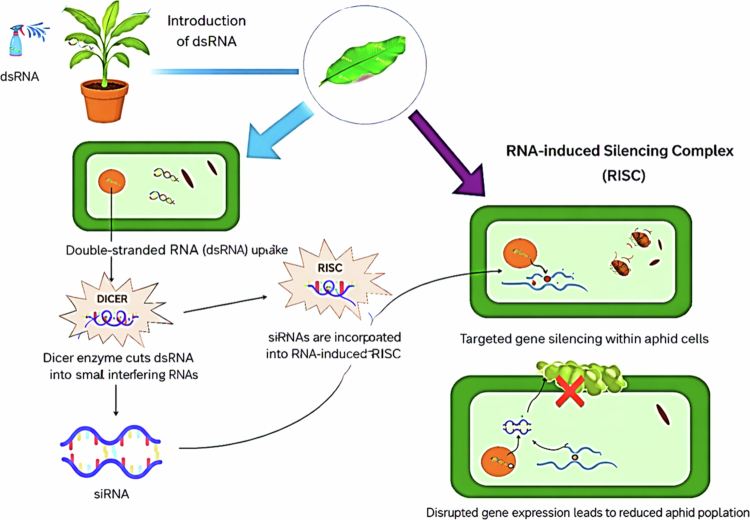
Using RNAi technique to control vectors for the control of viral diseases; dsDNA (Double stranded DNA) will be introduced; dsDNA will be sliced by DICED to produce fragments for RISC (RNA- induced silencing complex); RISC will produce the siRNA (small interference RNA) which would be consumed by the aphids; siRNA will alter the reproductive system of the aphid to control the population.

Banana bunchy top disease (BBTD) is a widespread viral disease affecting banana crops, caused by the Banana bunchy top virus (BBTV), a member of the genus *Babuvirus* in the family *Nanoviridae*. It is characterized by the narrowing and clustering of the leaves at the apical end, giving the plant a distinct bunched appearance. The main clinical symptoms include marginal chlorosis (yellowing), dark green streaks on petioles, and typical streaking of discrete dark green longitudinal dots and dashes on the midrib and large veins. Early identification of these symptoms is crucial for managing the disease. BBTV is primarily transmitted by the banana aphid (*Pentalonia nigronervosa*), which acquires the virus when feeding on infected plants and can then transfer it to healthy ones.^110^ The aphid's mobility facilitates local spread, while longer-distance spread usually occurs through the movement of infected planting materials such as suckers and corms. The disease causes severe stunting, significant yield loss, and reduced fruit quality, as well as infected trees rarely produce marketable fruit, and even this is often malformed. Currently, there is no immunity line in *Musa* against BBTV, making conventional breeding for resistance difficult.^111^ Since traditional methods have not been effective, developing resistant varieties is viewed as the most promising long-term solution. Recent advancements in biotechnology, particularly host-induced RNA interference (RNAi), offer new opportunities for enhancing plant resistance against BBTV and its vector (Figure 3).

RNAi-based methods involve silencing specific viral or vector genes to inhibit viral replication or aphid transmission.^112^ This technology has demonstrated success in controlling other plant viral and vector-borne diseases, and recent research further emphasizes its potential application for controlling BBTD and BBTV through sustainable methods targeting aphid vectors.

#### Xanthomonas bacterial wilt

Xanthomonas bacterial wilt of banana, also known as moko disease, is one of the most damaging bacterial pathogens affecting bananas, often causing an 80% reduction in yields or complete crop failure.^107^ *Ralstonia solanacearum* is the causative agent, a negative bacterium belonging to the *Proteobacteria phylum*, and is considered one of the most virulent plant pathogens worldwide. Early identification of symptoms is essential for preventing the spread of the pathogen and transmission by vectors.^113^ In large-scale production, disease spread is usually caused by infected pruning tools. The most common symptoms include foliar chlorosis, leaf wilting, fruit rot, and, most notably, discoloration of the vascular tissue in the pseudostem, rhizomes, and leaf sheaths, which change from light to dark brown, often accompanied by a slimy bacterial exudate on stem cross-sections.^114^ *Ralstonia solanacearum* exists in multiple genetic lineages, including the SFR (small, fluid, round) and A strains, spread by insect vectors, and the B strain, primarily transmitted *via* root-to-root contact or through infected planting material. Prompt diagnostic measures are necessary to prevent outbreaks before symptoms appear. Among advanced technologies, spectrometric measurements based on specific light wavelengths show promise for early detection of the disease in plant development, offering nondestructive, direct measurements of plant tissues.^115^ Genetic engineering is a promising approach to developing BXW-resistant banana varieties. Transgenic techniques have been used to introduce resistance genes from other crops into banana germplasm, thereby enhancing host resistance. Notably, transgenes such as hypersensitive response assisting protein (HRAP) and plant ferredoxin-like protein (PFLP) have been successfully expressed in cultivated bananas. These transgenic lines demonstrated complete protection against BXW in laboratory, greenhouse, and field trials, significantly limiting disease development and spread. Additionally, agronomic traits, including yield and flowering, are unaffected. Recent studies using CRISPR/Cas9 gene editing are exploring methods to target susceptibility genes, aiming to inhibit bacterial wilt susceptibility more quickly and accurately, providing a faster alternative to traditional breeding for disease resistance.^109^ Furthermore, genetic engineering addresses concerns related to gene flow and clonal propagation in bananas, offering a sustainable and safe method for crop improvement.

#### Banana streak virus

Banana streak virus (BSV) is part of the *Caulimoviridae* family, and the genus *Badnavirus* is one of the five main viruses affecting banana cultivation. BSV has been classified into four species: banana streaked OL virus (BSOLV), banana streak imo virus (BSIMV), banana streak Mysore virus, and banana streak Goldfinger virus (BSGFV). These agents cause banana streak mosaic disease, which has recently been identified as a major obstacle to banana production. The most common infection route occurs through banana plantlets produced *via* micropropagation and *in vitro* culture, which activate endogenous BSV sequences embedded in the banana genome. Recent research has successfully utilized the CRISPR/Cas9 system to disrupt these eBSV sequences in the B genome of “Gonja Manjaya” (AAB). By creating mutations at the targeted viral sites, the study produced plants that remained asymptomatic under stress conditions, overcoming a significant bottleneck in banana breeding and the distribution of B-genome hybrids.^31^ Additionally, climate change related to global warming, particularly shifts in water cycles and temperature in tropical regions, increases the risk of BSV emergence.^116^ Moreover, intensified breeding programs aimed at developing hybrid cultivars that are more disease-resistant and higher-yielding have also been linked to increased BSV prevalence. Key vectors responsible for spreading BSV include the mealybug species *Planococcus citri* and other *Pseudococcus* species. Phylogenetic studies show that BSV exists in two forms: an episomal form that actively infects plant cells and an integrated form where viral DNA is inserted into the banana genome.

#### Burrowing nematode

**Figure 4.**
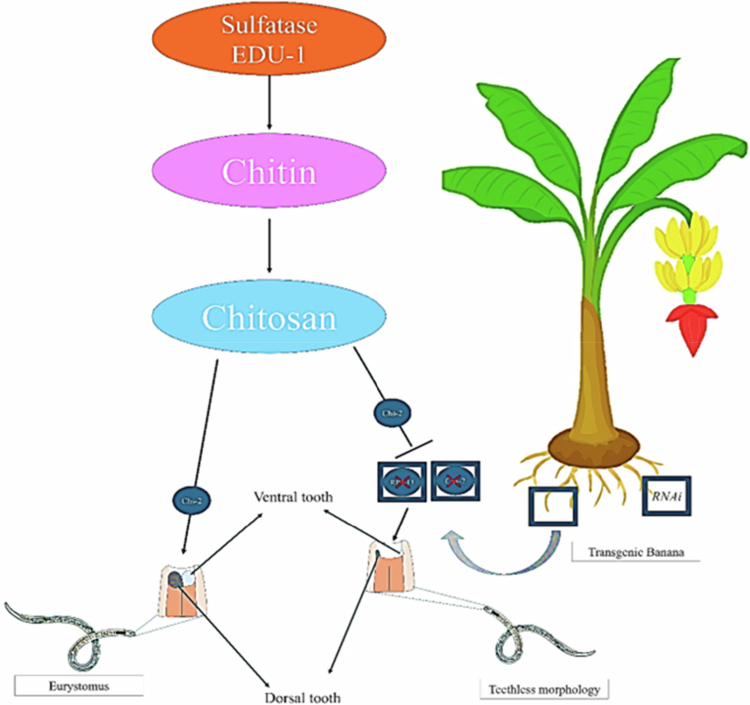
Introduction of RNAi to modify the teeth morphology of nematodes to reduce the damage. a. Under *chs-2* gene, the nematodes can develop dorsal strong teeth b. By the introduction of RNAi into the banana, the *chs-2* gene transcription will be arrested leading to teethless morphology in the further generations.

Banana burrowing nematode disease is caused by a nematode called *Radopholus similis*, which damages banana roots by causing necrosis and toppling. Traditional control with nematicides raises environmental and toxicity concerns and is often ineffective because the nematodes adapt, and most commercial banana cultivars lack natural resistance. Genetic engineering offers a promising alternative. Transgenic East African highland banana varieties have been developed using RNA interference (RNAi) technology, where the plants produce double-stranded RNA that targets specific nematode genes, such as *Rps13* and chitin synthase (*Chs-2*).^117^^,^^118^ This approach inhibits nematode reproduction, reduces root damage, and enhances plant health. Additional strategies include the use of nematode cysteine proteinase inhibitors, like rice cystatin, which can reduce nematode populations by up to 70% (Figure 4). Gene editing with CRISPR also holds promise by either knocking out susceptibility genes or introducing resistant genes without adding foreign DNA.^119^ Overall, these genetic engineering methods provide sustainable and effective solutions for managing banana burrowing nematode disease, addressing the limitations of traditional techniques and the lack of natural resistance in cultivated banana plantations. This technology is poised to stabilize banana production, a vital crop in many regions.

#### Cigar-end rot

Cigar-end rot in bananas is a necrotic disease primarily caused by *Trachysphaera fructigena*, although *Verticillium theobromae* may also sometimes cause it. Infection occurs in the fruit through the flower during very early stages of development, leading to focal necrosis at the tip of the fingers that resembles the burnt end of a cigar. The infection reduces fruit quality and is characterized by visible fruit rot that worsens during postharvest storage and transportation, resulting in significant market losses.^120^ Control methods rely on fungicides and cultural practices to reduce infection. Recently, advances in gene engineering offer a promising solution for the precise modification of susceptibility genes or the insertion of resistance genes via CRISPR/Cas9 editing without introducing foreign DNA.^121^ This technique provides permanent, highly specific resistance, which is especially valuable for the mostly asexual biotype cultivars, limited to traditional breeding. Biotechnological tools target pathogenic processes, enhance innate immunity, and promote sustainable disease control, thus minimizing losses and increasing banana production. Various genetic engineering approaches have shown promise in developing banana cultivars resistant to cigar-end rot, including overexpressing defense-related proteins, activating receptor kinases involved in immune signaling, and modifying genes involved in reactive oxygen species (ROS) metabolism.^122^ Moreover, genome-editing technologies such as CRISPR/Cas9 are increasingly used to accurately identify susceptibility genes and activate defense pathways linked to resistance. By integrating detailed molecular insights into disease resistance and conducting precise genome modifications, genetic engineering offers a strategic pathway to developing elite banana varieties resistant to cigar-end rot with minimal yield loss.^66^

### Future prospects and research directions

Recent breakthroughs in genomic resources, molecular tools, and transformation technologies provide greater opportunities to overcome past barriers in improving bananas. However, unlike seed-propagated crops, the future development of *Musa* spp. must specifically address major biological limitations such as polyploidy, clonal propagation, and parthenocarpy, which directly influence the importance of molecular cytogenetics and genome editing techniques. Polyploidy, especially the dominance of triploid genomes (AAA, AAB, ABB), presents significant challenges for cytogenetic analysis and genome manipulation. The way chromosomes pair, their structural rearrangements, and allele dosage effects complicate trait determination and gene function studies. Therefore, molecular cytogenetics strategies should include creating genome-specific probes and employing high-resolution methods like oligo-based FISH and long-read sequencing-based cytogenetics to accurately differentiate subgenomes and detect chromosomal variations. Concurrently, genome editing systems such as CRISPR/Cas need refinement to enable multiallelic targeting, allowing controlled modification of all gene copies to produce stable phenotypes in polyploid backgrounds. Although clonal propagation limits recombination and genetic diversity, it offers a unique advantage in banana improvement by enabling mass propagation of elite edited or transgenic lines without segregation. Nevertheless, this approach requires careful monitoring of genetic stability, as tissue culture may induce somaclonal variation and chromosomal aberrations. Molecular cytogenetic techniques like FISH and GISH, along with high-throughput molecular markers, will be crucial for ensuring clonal fidelity and detecting genome instability. Emerging methods such as single-cell genomics and epigenetic profiling can further enhance quality control in clonal propagation systems. Parthenocarpy and other forms of sterility remain significant breeding challenges in bananas, limiting the ability to develop segregating populations for linkage mapping and traditional genetic analysis. This hampers trait mapping and slows genetic progress. Addressing this requires prioritizing diploid progenitors, induced fertile lines, somatic hybridization, and association mapping within germplasm panels from various sources. Molecular cytogenetics can also help identify chromosome regions linked to sterility and parthenocarpy, enabling targeted interventions via genome editing. Notably, these biological constraints also create opportunities for precise breeding. Combining genome editing with cytogenetic validation allows targeted modification of genes, repetitive DNA sequences, and structurally complex loci that are inaccessible through traditional breeding. Additionally, endogenous viral elements like banana streak virus (BSV) highlight the importance of cytogenetic methods for monitoring genome activity and stability, especially under stress and during tissue culture. Integrating multiomics approaches such as transcriptomics, proteomics, metabolomics, and epigenomics with machine learning-based predictive models will deepen understanding of the regulation of complex traits like stress tolerance, yield, and fruit quality. When combined with cytogenetic data, these tools can accelerate the identification of candidate genes and pathways critical for banana improvement.

Higher drought-induced activation of aquaporin genes such as MaPIP1;1 highlights the potential of targeting water channel regulation to enhance dehydration tolerance. Future research could explore genome editing and promoter engineering strategies to fine-tune AQP expression, enabling plants to maintain optimal hydraulic conductivity even under severe water limitation. Similarly, the improved tolerance observed in *Musa*DHN1-overexpressing lines—characterized by elevated proline accumulation and reduced lipid peroxidation—suggests that dehydrin-mediated osmoprotection can be harnessed more broadly across banana genotypes. Subsequent studies should focus on stacking multiple osmoprotective genes or integrating DHN-based traits with ion-homeostasis pathways to further strengthen salinity resilience in high-yielding commercial cultivars. Further, the coordinated upregulation of thermo-protective genes such as HSP70, HSP90, and DNAJ under heat stress, coupled with the downregulation of their corresponding miRNA regulators, underscores the significance of the miRNA–HSP regulatory axis in banana thermotolerance. In Future, efforts should prioritize dissecting these regulatory networks across developmental stages and diverse stress intensities. Leveraging CRISPR-based miRNA modulation or identifying naturally heat-resilient alleles can accelerate the breeding of cultivars capable of withstanding rising global temperatures.

## Conclusion

To summarize, genetic enhancement of bananas is progressing rapidly through the combination of classical breeding, molecular markers, and the latest genome editing strategies. However, the success of these methods depends on how well they are adapted to the unique biological features of bananas, especially polyploidy, clonal propagation, and parthenocarpy. These factors not only limit natural breeding but also influence how new technologies like molecular cytogenetics and CRISPR-based editing should be applied. Shifting focus to banana-specific improvement strategies will help address these challenges, combining cytogenetic tools with genomic and biotechnological solutions to manage genome complexity, preserve clonal fidelity, and enable precise genetic modifications. The ability to target multiple alleles in polyploid genomes, maintain genetic stability in clonal systems, and overcome sterility issues will be crucial for future success. Emerging challenges such as Fusarium wilt (TR4) and climate change continue to emphasize the need for resistant banana varieties. Achieving this goal will rely on sustained global cooperation, efficient utilization of worldwide germplasm resources, and the integration of advanced technologies tailored to *Musa* biology. Despite ongoing concerns, the combined approach of cytogenetics, genomics, and genome editing offers a promising pathway toward sustainable banana improvement.
